# Chronic consumption of fructose rich soft drinks alters tissue lipids of rats

**DOI:** 10.1186/1758-5996-2-43

**Published:** 2010-06-23

**Authors:** Jose D Botezelli, Rodrigo A Dalia, Ivan M Reis, Ricardo A Barbieri, Tiago M Rezende, Jailton G Pelarigo, Jamile Codogno, Raquel Gonçalves, Maria A Mello

**Affiliations:** 1 São Paulo State University - UNESP, Department of Physical Education, Av: 24-A, 1515 Bela Vista, Zip code: 13506-900, Rio Claro - São Paulo- Brazil

## Abstract

**Background:**

Fructose-based diets are apparently related to the occurrence of several metabolic dysfunctions, but the effects of the consumption of high amounts of fructose on body tissues have not been well described. The aim of this study was to analyze the general characteristics and the lipid content of different tissues of rats after chronic ingestion of a fructose rich soft drink.

**Methods:**

Forty-five Wistar rats were used. The rats were divided into three groups (n = 15) and allowed to consume water (C), light Coca Cola ^® ^(L) or regular Coca Cola^® ^(R) as the sole source of liquids for eight weeks.

**Results:**

The R group presented significantly higher daily liquid intake and significantly lower food intake than the C and L groups. Moreover, relative to the C and L groups, the R group showed higher triglyceride concentrations in the serum and liver. However, the L group animals presented lower values of serum triglycerides and cholesterol than controls.

**Conclusions:**

Based on the results, it can be concluded that daily ingestion of a large amount of fructose- rich soft drink resulted in unfavorable alterations to the lipid profile of the rats.

## Background

Fructose is a monosaccharide commonly found in the contemporary Western diet. Ingestion of large amounts of fructose is frequently related to the consumption of fast foods and soft drinks. The sharp rise in the incidence of obesity and metabolic syndrome in the United States that has occurred in recent years has been correlated with a 30% increase in the population's total fructose ingestion that has occurred over the same time frame; this increase is partly due to the introduction of fructose-rich corn syrup as the major sweetener in soft drinks and other foods [1-3]. In animal models, fructose-based diets have been shown to be related to a variety of metabolic dysfunctions, many of which relate to lipid metabolism [3]. These metabolic dysfunctions are associated with increased serum levels of free fatty acids, triglycerides and very low density lipoprotein (VLDL) and with hypertension, insulin resistance, hyperinsulinemia, hyperglycemia and obesity [4-7]. Changes of this type can result in the metabolic syndrome [8], which is characterized by an increased risk of cardiovascular and circulatory disturbances.

Unlike glucose, which is metabolized in every body tissue, fructose is primarily metabolized in the liver and poorly stimulates insulin and leptin secretion [9,10]. While the metabolism of glucose is negatively regulated by phosphofructokinase, fructose can continually enter the glycolytic pathway, leading to a high production of pyruvate and providing hydrocarbons for glycerol and triglyceride synthesis. The resulting excess in intracellular energy flux is associated with insulin resistance, stimulation of inflammatory pathways, ApoB production, and uncontrolled triglyceride synthesis, which together lead to hepatic stress [11,12]. Using animal models, Kelley et al. [13] demonstrated that the increase in triglyceride synthesis and the decrease in VLDL clearance that occur after ingestion of high amounts of fructose are associated with an increase in serum lipid levels. Increased levels of circulating lipids stimulate the uptake of triglycerides by muscles and other tissues and can lead to a state of insulin resistance in these tissues [14]. Additionally, alterations in the activities of liver enzymes, especially pyruvate dehydrogenase, change insulin receptor phosphorylation and increase inflammatory cytokine production [12]. In rats fed for two weeks with a fructose-rich diet (66% of calories from fructose), Catena et al. [15] demonstrated that insulin receptor mRNA levels, as well as the number of insulin receptors in muscle and liver, were significantly lower than in rats fed a balanced diet. Several studies have demonstrated a correlation between chronic consumption of fructose-rich soft drinks and the presence of symptoms of non-alcoholic fatty liver disease [16,17]; these effects were correlated with the liver lipogenic effect of fructose [14]. Although the consequences of the consumption of fructose-based diets have been extensively discussed, there is little information about the particular effects of such diets on specific tissues [18].

The aim of this study was to analyze the general characteristics and the lipid content of specific tissues of Wistar rats that consumed a fructose-rich soft drink for eight weeks. For comparison, rats that consumed a soft drink low in fructose (light soft drink) were also analyzed for the same period.

## Methods

### Animals

Forty-five weaned male Wistar rats, 21 days of age, obtained from the Central Biotherium of UNESP-São Paulo State University, Botucatu Campus, were used. During the experiment, the animals were kept in collective polyethylene cages (5 rats per cage) in a closed biotherium at 25°C under a light/dark photoperiod of 12/12 hours. The animals received commercial chow for rodents (Labina of Purina^®^) *ad libitum*. Body weight, as well as food and liquid intake per cage, was measured and recorded once a week. All experiments involving animals were reviewed and approved by the Ethics Committee of The Herminio Ometto Foundation (UNIARARAS) Case Number: 068/2008

### Experimental groups

The animals were divided randomly into three groups (n = 15 per group) according to the liquid source utilized. Control Group (C) animals ingested water *ad libitum *as the only liquid source; Light soft drink Group (L) animals ingested the soft drink Coca Cola Light^® ^(The Coca-Cola Company, Atlanta, GA) *ad libitum *as the only liquid source; and Regular soft drink Group (R) animals ingested the soft drink Coca Cola^® ^(The Coca-Cola Company, Atlanta, GA) *ad libitum *as the only liquid source. The soft drinks offered to the animals had the carbon dioxide removed by sonication in a small container.

The composition of the regular and light soft drinks is described in Table 1.

**Table 1:** Soft drink composition* in g and kcal.

	L (200 ml)	R (200 ml)
Energetic Value (Kcal)	0	85

Carbohydrates (g)	0	21

Lipids (g)	0	0

Sodium (mg)	23	10

L = Light Soft Drink; R = Regular Soft Drink.

*According to Manufacturer

### Evaluations

An oral glucose tolerance test (OGTT) was performed on each animal forty eight hours before the sacrifice. At the beginning of the test, a first blood collection from a cut at the tip of the tail was performed (time zero). Later, a polyethylene catheter was orally introduced into the stomach, and a 20% glucose solution (2 g/kg of body weight) was administered. Blood samples were collected at 30, 60 and 120 minutes after glucose administration using heparinized capillaries calibrated to 25 μL for glucose measurement by the glucose oxidase method [19]. The glucose concentrations during the OGTT were evaluated from the total areas under the serum glucose (AG = mg*120 min) curve using the trapezoidal method with ORIGIN PRO 8 software.

At the end of eight weeks, the animals were sacrificed by decapitation for tissue and blood sample collection. The blood was centrifuged at 1700 rcf rpm for 10 minutes to separate the serum. Analysis of serum glucose levels was determined by the glucose oxidase method [19]. Serum triglycerides (TG), total cholesterol, High Density Lipoprotein-Cholesterol and Low Density Lipoprotein-Cholesterol were analyzed by means of colorimetric enzymatic methods (Laborlab kits). Serum free fatty acids (NEFA) were measured by a modification of the Regow et al. [20] method as described in Nogueira et al. [19].

Liver and soleus muscle samples were collected for the evaluation of triglyceride concentrations. Adipose tissue was extracted from the retroperitoneal, mesenteric and posterior subcutaneous regions [21] for weighing and triglyceride concentration determination. For triglyceride content analysis, the tissue samples were placed in tubes containing 0.5 ml of 0.1% Triton X-100, sonicated for 45 seconds, and centrifuged at 1700 rcf for 10 minutes. The supernatant was saved for the determination of the triglyceride level, according to the method described by Nogueira et al. [19].

### Statistical Treatment

The results are expressed as the mean ± standard deviation. For statistical treatment, one-way ANOVA was used, followed, when appropriate, by the Newman Keuls *post hoc *test with the assistance of Statistica 7.0^® ^software. In every case, the significance level was set at 5%.

## Results

**Figure 1:**
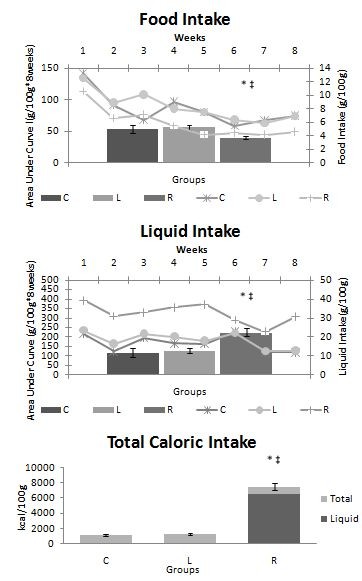
**Daily food and liquid intake**. Daily food, liquid and caloric intake and area under the curve of food and liquid intake during the experiment. Food and water intake were measured and registered once a week. Caloric intake was estimated from the food intake. The results were analyzed by the calculated areas under the curves of food and liquid intake. C = Control; L = Light Soft Drink; R = Regular Soft Drink. n = 15 animals per group. * ≠ C. ‡ ≠ L.

**Figure 2:**
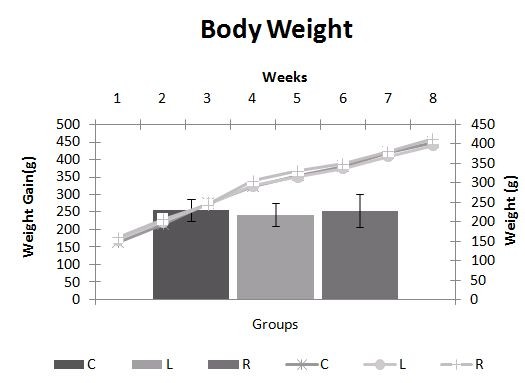
**Change in body weight**. Evolution of body weight and weight gain of rats during the experiment. Body weight was measured and registered once a week. C = Control; L = Light Soft Drink; R = Regular Soft Drink. n= 15 animals per group. * ≠ C. ‡ ≠ L

As shown in Figure 1, the R group animals presented a mean daily food intake that was significantly lower and a mean liquid intake that was significantly higher than those of the animals in the C and L groups. There was no significant difference in the evolution of body weight among the three groups of animals during the experiment (Figure 2).

**Figure 3:**
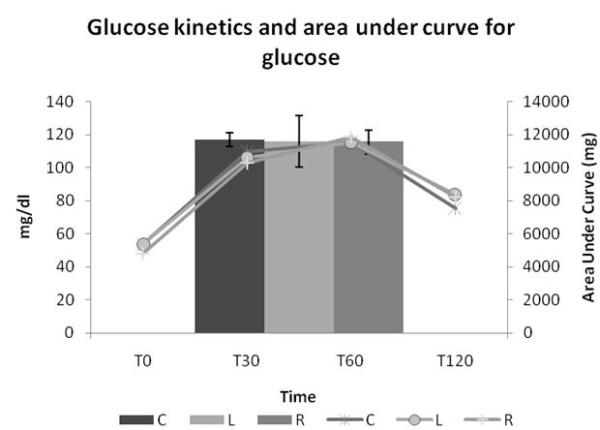
**Glucose kinetics and area under curve of glucose during OGTT**. Glucose kinetics and area under the curve of blood glucose levels during the glucose tolerance test. A 20% glucose solution (2 g/kg of body weight) was administered to the rats by gavage, after a 15 h fasting. Blood samples were collected before and at 30, 60 and 120 minutes after glucose administration. The results were analyzed by the calculated areas under the curves of serum glucose. C= Control; L= Light Soft Drink; R = Regular Soft Drink. n = 15 animals in each group.

Figure 3 shows the glucose levels and area under the curve of these levels measured during the OGTT. No difference was observed among the groups.

Table 2 shows the differences in serum glucose, insulin, triglyceride, AGL, total cholesterol, HDL and LDL levels among the groups. There was a significant increase in serum glucose in the L group compared to the C group. The R group animals exhibited higher circulating triglyceride concentrations than animals in either of the other groups. In the L group, there was a significant reduction in total serum cholesterol compared to controls. L group animals also showed a significant decrease in serum LDL cholesterol levels compared to both C and R group animals.

**Table 2:** Serum Glucose (mg/dl), Triglycerides (mg/dl), NEFA (mEq/dl), Total Cholesterol (mg/dl), HDL (mg/dl) and LDL (mg/dl) at the end of the experiment.

	C	L	R
Glucose	117 ± 15	138 ± 14*	129 ± 14

Triglycerides	229 ± 21	190 ± 57	326 ± 80*^‡^

NEFA	642 ± 232	582 ± 272	792 ± 427

Total Cholesterol	98 ± 13	77 ± 18*	90 ± 19

LDL Cholesterol	51 ± 7	33 ± 12*^│^	49 ± 13

HDL Cholesterol	41 ± 4	41 ± 5	39 ± 3

C = Control; L = Light Soft Drink; R = Soft Drink. n = 8 animals in each group.

*≠C

‡≠L

│≠R

**Figure 4:**
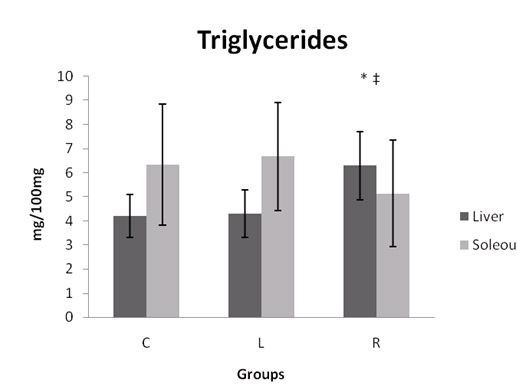
**Liver and soleus triglycerides**. Liver and soleus muscle triglycerides of the rats at the end of the experiment. C = Control; L = Light Soft Drink; R = Soft Drink. n = 8 animals in each group. * ≠ C. ‡ ≠ L.

In Figure 4, it can be observed that the R group presented significantly higher liver triglycerides than the C and L groups.

Table 3 shows the weight and triglyceride concentration of adipose tissue of the subcutaneous, retroperitoneal and mesenteric regions of the three groups of animals. In the R group, there was a significant increase over both of the other groups in the weight of mesenteric and retroperitoneal adipose tissue. No differences were observed in triglycerides concentrations among the groups.

**Table 3:** Weight (g/100 g) and Triglycerides content (mg/100 mg) of Adipose Tissue of the different regions at the end of the experiment

	C	L	R
	**Weight**		
Subcutaneous	0.3 ± 0.1	0.4 ± 0.1	0.4 ± 0.1
Retroperitonial	0.4 ± 0.1	0.4 ± 0.1	0.7 ± 0.1*‡
Mesenteric	0.6 ± 0.1	0.6 ± 0.1	0.8 ± 0.2*‡

	**Triglyceride content**		
Subcutaneous	15.2 ± 3.01	14.5 ± 3.4	14.2 ± 3.0
Retroperitonial	14.1 ± 3.2	14.5 ± 2.7	12.8 ± 3.4
Mesenteric	19.1 ± 5.2	19.9 ± 4.2	15.7 ± 3.4

Results expressed as means + standard deviation. C = Control; L = Light Soft Drink; R = Regular Soft Drink. n = 8 animals of each group.

*≠C

‡≠L

## Discussion

This study determined the effects of chronic ingestion of fructose-rich and low-fructose soft drinks on the general characteristics and the lipid profile of rats. *Ad libitum *consumption of fructose-rich soft drink was significantly higher than of water or of light soft drink and affected food intake, resulting in significantly lower food consumption in the R group animals. Nevertheless, this high consumption of fructose-rich soft drink did not affect body weight of the rats. A similar result was obtained in another study [22] and our results corroborate the finding that short-term effects of fructose are not correlated with body weight [22].

The light soft drink used in this study is poor in fructose but rich in sweetener (aspartame). There is evidence in the literature suggesting that ingestion of artificial sweeteners increases food intake by animals and humans [23,24]. The literature is inconsistent with respect to the effects of artificial sweeteners on body weight; some studies report an increase in body weight of animals after ingestion of artificial sweeteners [25-27], while others do not report any alteration [28,29]. In this study, the consumption of light soft drinks did not significantly affect either food intake or body weight.

The caloric intake of the R group animals in this study was approximately four times higher than that of the L and C group animals. However, there was no significant difference in body weight. It is known that, in humans, caffeine intake at a dose of 4 mg/kg every two hours alters both the basal metabolic rate (increasing it between 8 and 11%) and the renin-angiotensin system [30-32]. The caffeine intake of the group R animals corresponding to 4.8 mg/kg every two hours was probably sufficient to offset the potential gain in weight that would otherwise have resulted from their increased caloric intake. Nevertheless, it should be noted that these animals ingested about five times more liquid than did the animals in the other two groups. Changes in the renin-angiotensin system may have been responsible for the large intake of regular soft drink by group R animals during the experiment. By comparison, the L group animals had an average caffeine intake of about corresponding to 1.02 mg/kg every two hours. This amount was probably not sufficient to cause changes in basal metabolism or in the renin-angiotensin system [30,32].

Even more, fructose fails to trigger a postprandial insulin secretion increase. The lack of insulin secretion in response to fructose in turn reduces leptin production from adipose tissue, which negatively alters central nervous system perception of energy stores [3]. This also might have influenced liquid intake of the R group in the present study. Araújo et al. [33] performed behavioral test in mice, in which they compared normal and sweet-blind mice in their preference for sucrose solutions and noncaloric sweetener sucralose solutions. In those tests, the sweet-blind mice showed a preference for calorie-containing sugar water that did not depend on their ability to taste, but on the caloric content of the beverage. In the analyzing the brains of the sweet-blind mice, these authors showed that the animals' reward circuitry was switched on by caloric intake, independent of the animals' ability to taste. The analyses revealed that the brain levels of dopamine, known to activate the brain reward circuitry, increased with caloric intake. Also, electrophysiological evaluations in this same study demonstrated that neurons in the nucleus accumbens, the food-rewards region, were activated by caloric intake, independent of taste

Glucose tolerance was analyzed using the OGTT; no differences among animals in the different experimental groups were observed in the area under the curve of serum glucose during the test. This finding is in agreement with the results of previous studies [34]. However, it is, important to notice that insulin secretion by pancreatic islet of fructose rich fed rats is increased, as previously reported by us [35] and other groups [36], probably increased insulin secretion kept serum glucose at control levels during the test.

Basciano et al. [37] showed that when the metabolic pathways of animals undergoing fructose overload are strictly investigated, both positive and negative effects of fructose consumption can be demonstrated. Although fructose is a potent regulator of glycogen synthesis and of glucose utilization by the liver, its regulatory effects decrease with chronic ingestion. In addition, because of its lipogenic properties, excess fructose in the diet makes the absorption of glucose difficult and elevates circulating TG and cholesterol.

Ingestion of fructose-based diets is characterized by metabolic dysfunctions and rapid increases in serum TG [4-7]. Our results bear out these findings; the highest values of serum TG were found in R group animals, and the difference was significant in comparison to the L group. Moreover, R group animals also had significantly higher liver and muscle TG concentrations than did C and L group animals.

After ingestion, fructose is absorbed in the intestine by the glucose transporter GLUT5. GLUT 5 receptors most important site of actions is the liver, where fructose is rapidly absorbed from the portal blood. In the liver fructose is converted to fructose-1-phosphate and enters the glycolytic pathway beyond the phosphofructokinase step, the main regulatory step of glycolysis. The phosphofructokinase activity responds to changes in glycogen stores and products of glycolysis, such as citrate and ATP and thus regulates glucose metabolism tightly. In contrast, fructose freely enters the glycolytic pathway and its metabolism leads to an accumulation of intermediates of glycolysis that are converted to glycerol and acetyl-coenzyme A (CoA) before being synthesized into fatty acids, very-low-density lipoproteins, and triglycerides [38]. Studies of acute fructose ingestion by fructose fed rats show a significant immediate increase in circulating triglycerides in comparison to balanced diet fed rats [39]. In addition to the increase in lipogenesis, fructose decreases the rate of lipid oxidation [40], which could explain, at least in part, the great visceral fat accumulation found both in the retroperitoneal and mesenteric adipose tissue and in the livers of R group animals.

It has been demonstrated that excessive ingestion of fructose can cause not only liver TG accumulation but also accumulation of inflammatory substances such as TNF-α, among others [41,42], and that the sum of these factors can contribute to hepatic insulin resistance and glucose intolerance [36] as well as to the development of a state of non-alcoholic hepatic steatosis.

The total cholesterol data in our study contrasts with other data presented in the literature [43], in which high levels of total cholesterol were found in rats that consumed fructose in excess. In our study, there was no difference in the total cholesterol levels of animals in the C group and those in the group R; however, group L animals showed reduced levels of total cholesterol compared with the C group. Soft drinks are highly caloric products and are frequently associated with the development of obesity [44]. In a recent review, Gaby [43] points out that high fructose consumption is often associated with very high levels of triglycerides and LDL in the bloodstream. In the present study, rats given soft drink without the addition of fructose (L group animals) presented lower values of serum LDL than those given soft drinks rich in fructose. The L group's LDL values were, in fact, lower than those of the control group. This difference may have been caused by the action of caffeine in increasing lipolysis and acting as an anti-obesity agent [45]. However, this hypothesis does not explain the observation that the R group did not present differences in LDL levels compared to the C group.

In summary, the rats that ingested fructose-rich soft drinks presented a significantly higher daily liquid intake and a significantly lower food intake than both control rats and those that ingested a light soft drink. Also, they had higher amounts of triglyceride both in serum and in the liver.

## Conclusions

Based on the results obtained in this study, it can be concluded that the daily ingestion of large amounts of fructose-rich soft drink by rats can lead to unfavorable alterations in serum lipid profiles and tissues lipid content. Such alterations were not observed with increased ingestion of soft drink low in fructose (light soft drinks). Therefore, the defects observed in the lipid profile are probably associated with the high fructose content of the soft drink.

## List of Abbreviations

ApoB: Apolipoprotein B; HDL: High Density Lipoprotein; LDL: Low Density Lipoprotein; NEFA: Non-esterified fatty acid; TG: Triglycerides; TNF-α: Tumor necrosis factor alpha; VLDL: Very Low Density Lipoprotein.

## Competing interests

The authors declare that they have no competing interests.

## Authors' contributions

JDB, RAD and IGMR were responsible for experimental design, data collection, statistical analysis and preparation of the manuscript. RAB, TMR and JGP were responsible for experimental design and data collection. JC and RG were responsible for collecting data and preparing the manuscript. MARM was responsible for experimental design, coordination of research and preparing the manuscript. All authors read and approved the manuscript.
